# Recent advances in PCR-free nucleic acid detection for SARS-COV-2

**DOI:** 10.3389/fbioe.2022.999358

**Published:** 2022-10-07

**Authors:** Xiaowei Ma, Jingsong Xu, Fei Zhou, Jing Ye, Donglei Yang, Hua Wang, Pengfei Wang, Min Li

**Affiliations:** Department of Laboratory Medicine, Institute of Molecular Medicine, Shanghai Key Laboratory for Nucleic Acid Chemistry and Nanomedicine, Renji Hospital, School of Medicine, Shanghai Jiao Tong University, Shanghai, China

**Keywords:** SARS-CoV-2, PCR-free detection, nucleic acids, CRISPR, isothermal amplification

## Abstract

As the outbreak of Coronavirus disease 2019 (COVID-19) caused by severe acute respiratory disease coronavirus 2 (SARS-COV-2), fast, accurate, and economic detection of viral infection has become crucial for stopping the spread. Polymerase chain reaction (PCR) of viral nucleic acids has been the gold standard method for SARS-COV-2 detection, which, however, generally requires sophisticated facilities and laboratory space, and is time consuming. This review presents recent advances in PCR-free nucleic acid detection methods for SARS-CoV-2, including emerging methods of isothermal amplification, nucleic acid enzymes, electrochemistry and CRISPR.

## 1 Introduction

The COVID-19 outbreak, which was caused by SARS-COV-2, started in Wuhan, China in 2019 and has lasted for more than 2 years (Wu et al., 2020a). According to WHO statistics, SARS-COV-2 has infected more than five billion people and caused 6 million deaths by the end of May 2022 (Who Health Organization, 2020). Meanwhile, studies showed that SARS-CoV-2 reinfection increased the hospitalization and mortality compared to the first infection (Alotaiby et al., 2022; Comba et al., 2022), which means that it is important to maintain long-term monitoring of this virus.

Due to the high transmissibility characteristic of SARS-COV-2, rapid and accurate diagnostics methods are needed to prevent the virus from spreading. PCR is a reliable and widely used gold standard method in clinical and research laboratories around the world (Lan et al., 2020). PCR exhibits excellent specificity and sensitivity in virus detection. However, the technique needs to be performed in a laboratory with expensive equipment, qualified clinical laboratory personnel, and a clean environment to avoid contamination. Therefore, the technology cannot operated at the point of care and is less cost-effective (Yadav et al., 2021). Moreover, the standard real-time-polymerase chain reaction (RT-PCR) protocol requires 2–3 h to complete. Limited by the time of sample collection, transportation, and manipulation, the final diagnosis result cannot be obtained within 4–6 h. Other detection methods also have some limitations (Table 1), such as antigen tests are less accurate when used in people with no symptoms (Khandker et al., 2021). Therefore, there is an urgent need to develop fast, accurate, and cost-effective methods for SARS-COV-2 diagnosis. In this review, we summarized the recent reports on PCR-free detection methods for SARS-COV-2.

**TABLE 1:** Current detection methods for SARS-COV-2.

Detection method	Biomarker	Advantages	Disadvantages
RT-PCR	Viral RNA	High sensitivity and specificity (gold-standard for diagnosis)	Expensive equipment require, time consuming
ELISA	Antigen/Antibody/Viral RNA	High sensitivity	Time consuming
Lateral Flow Assay (LFA)	Antigen/Antibody/Viral RNA	Low-cost and rapid detection	Low sensitivity and specificity
CT	X-ray Imaging feature	reflect the severity of the disease	Expensive equipment require, low sensitivity and specificity for virus diagnosis

## 2 Isothermal amplification methods for SARS-COV-2

### 2.1 Reverse transcription loop‐mediated isothermal amplification

Reverse Transcription Loop‐Mediated Isothermal Amplification (RT-LAMP) systems are the most commonly used isothermal amplified nucleic acid detection methods and have the characteristics of speediness, high sensitivity and high specificity (Notomi et al., 2000; Chaouch, 2021). As regards SARS-CoV-2 detection, RT-LAMP is considered as an ideal alternative to RT-PCR. Lots of LAMP-based systems have been developed and some of them have been used to prevent further spread of the SARS-COV-2 pandemic (Andryukov et al., 2021).

The RT-LAMP detection systems are based on strand displacement DNA polymerase and 4-6 primers (Parida et al., 2004; Wong et al., 2018). Technically, two inner primers and two outer primers are designed to recognize the particular regions in the target sequence, while two extra loop primers are used to accelerate amplification and maintain stability (Chaouch, 2021). The limit of detection (LoD) of one step RT-LAMP is 10 copies of RNA fragments within 40 min at 65°C and the test results can be visualized by agarose gel electrophoresis, turbidity, fluorescence or colorimetry (Yuan et al., 2020). Researchers from different countries have done a number of clinical tests to study the detection performance of RT-LAMP. Results showed that it had excellent specificity, while the sensitivity fluctuated between 87% and 98% (Lee et al., 2020a; Broughton et al., 2020; Ganguli et al., 2020; Jiang et al., 2020; Anahtar et al., 2021). To further increase the sensitivity and reduce the contamination, several studies focused on the improvement of one step RT-LAMP. For example, replacing the universal transport medium with saline and adding an upfront RNase inactivation step can raise the sensitivity (Anahtar et al., 2021). Designing special primers for fluorogenic oligonucleotide strand exchange (OSD) probes can allow multiple genomic targets of SARS-CoV-2 included in one assay (Lamb et al., 2020). Based on the optimization of RT-LAMP, the Id NOW™ COVID-19 assay (Abbott Laboratories) has been approved by FDA with Emergency Use Authorization (EUA) (Yuan et al., 2020). This method targets the SARS-CoV-2 RdRp gene and can show positive results in high concentration samples within 5 min. However, one step RT-LAMP systems are still mostly used as lab-centric diagnostic techniques (Song et al., 2017; Pang et al., 2020). In order to meet the actual application requirements, many researchers chose to combine RT-LAMP with other innovative techniques. Researchers from China devised a diagnostic method based on RT-LAMP and nanoparticle-based lateral flow biosensor (LFB) (Zhu et al., 2020). In this reaction system, primers are labeled by FITC (fluorescein)-digoxin and biotin, so the results can be seen by naked eyes through immunoreactions and biotin/streptavidin interaction. The LoD is 12 copies per reaction and the sensitivity and specificity were both 100% (Zhu et al., 2020). Some experts believe this technology can be a useful diagnostic tool for resource-poor regions (Chaouch, 2021).

**FIGURE 1:**
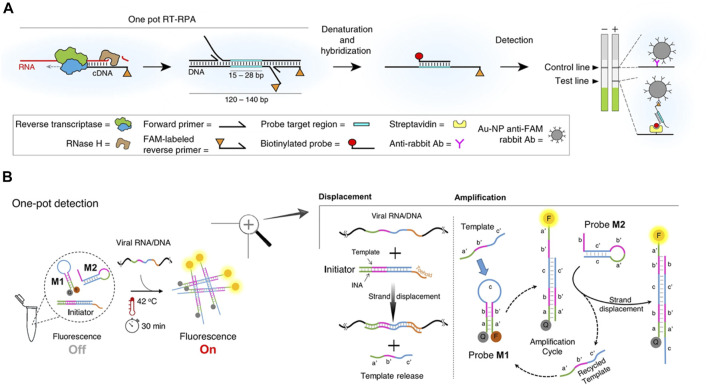
Schematic of Isothermal Amplification methods for SARS-COV-2. **(A)** eRPA assay for detection of SARS-COV-2. Viral RNA is first copied to cDNA by RT, then degraded by RNase H. The cDNA product is amplified by RPA using a forward and a FAM-labeled reverse pair of primers specific to the target sequence. The amplified material is then denatured and hybridized to a biotinylated probe. Dual FAM-labeled and biotin-labeled products are detected on lateral flow strips. Source data are available in the Source Data file. Reprint from (Qian et al., 2020), copyright (2020), with permission from Springer Nature. **(B)** NISDA assay for rapid detection of SARS-CoV-2 RNA. The reaction mixture contains three key components, including a DNA duplex (the Initiator), and two DNA molecular beacon structures (probe M1 and probe M2). In presence of viral RNA/DNA, toehold-mediated template displacement and cascade signal amplification occur sequentially, following by fluorescence detection after 30 min at 42 °C. INA is intercalating nucleic acid with enhanced binding affinity. F and Q denote for 6-Fam fluorophore and bhq-1 quencher, respectively. Letter labels denote for domains. The domains labeled with primes are complementary sequences. Reprint from (Mohammadniaei et al., 2021), copyright (2020), with permission from Springer Nature.

Although most of these have not yet been optimized for SARS-CoV-2, they have potential applications in the future. Digital RT-LAMP can realize absolute quantification of target sequences and offers a flexible test way for common laboratories (Lin et al., 2019). Rolling circle amplification (RCA) is a linear signal amplifying mechanism. RCA-LAMP can significantly improve the detection sensitivity and the reaction efficiency (Tian et al., 2019). Using naphthoquinone-imidazole (NQIM) probes to couple with LAMP can realize DNA amplification in 10 min (Chen et al., 2019). In summary, RT-LAMP-based technology has a great advantage in specificity and detection speed. In terms of sensitivity, it is reported to be one to two times less than RT‐PCR (Jiang et al., 2020; Wang et al., 2022a). The complexity of primer design is an inevitable drawback of RT-LAMP (Figure 1), but it is still a promising assay due to its compatibility for many other technologies (Qian et al., 2020; Chen et al., 2020).

### 2.2 Reverse transcription recombinase polymerase amplification

Owing to its low operation temperature (25–42°C) and freeze-dried reagents, recombinase polymerase amplification (RPA) is undoubtedly a promising isothermal molecular technique for pathogen detection (Daher et al., 2016; Esbin et al., 2020). RPA entails two primers with simple design requirements compared to LAMP. It uses the *Escherichia coli* RecA recombinase and single-strand DNA binding protein (SSB) to substitute the heat denaturation step (Piepenburg et al., 2006). Meanwhile, DNA polymerase, usually from *Staphylococcus aureus,* is used to extend the chain. Polyethylene glycol or Carbowax20M initiates the reaction and creatine kinase generates ATP for the system (Piepenburg et al., 2006; Li and Macdonald, 2015). As for RNA targets, reverse transcriptase can realize one-step RT-RPA operation in 20 min (Hill-Cawthorne et al., 2014). RPA can tolerate common nucleic acid amplification inhibitors and can operate normally with various sample types, such as serum (Teoh et al., 2015), stool (Amer et al., 2013), nasal (Boyle et al., 2014) and even milk (Santiago-Felipe et al., 2015). Because of the advantages mentioned above, RPA has been used for the detection of a wide range of pathogens and cancers and has led to a number of post-detection methods (Amer et al., 2013; Loo et al., 2013). For example, hybridization assays can be realized when combined with ELISA (Santiago-Felipe et al., 2014). When combined with LAMP, it can be 10 times more sensitive than qPCR (Yuan et al., 2020). The limit of detection and turnaround time varies between amplicon size, assay and primers.

RPA-based detection technology of SARS-CoV-2 ensures accuracy while being faster and more portable than other current technologies (Wu et al., 2020b). Because of using simple primers, RPA makes it easier to achieve simultaneous detection of multiple pathogens or multiple targets, which further improves detection accuracy (Yuan et al., 2020). Ahmed et al. developed three RT-RPA assays targeting the RNA-dependent RNA polymerase (RdRP), envelope protein (E), and nucleocapsid protein (N) genes of SARS-CoV-2 (El Wahed et al., 2021). This method can detect three targets simultaneously at 42°C for 15 min and the entire reaction system is integrated into a mobile suitcase. The LoD of this assay is two RNA molecules for the RdRP gene and 15 RNA molecules for E and N genes (El Wahed et al., 2021). As for clinical specimens, the sensitivity and specificity in comparison to RT-PCR are 94% and 100% for RdRP gene, 65% and 77% for E gene; and 83% and 94% for N gene (El Wahed et al., 2021). This RPA-based technology can contribute to assisting the detection of SARS-CoV-2 in low-resource areas such as Africa. Another RPA-based assay is combined with CRISPR-Cas12a. Ding et al. (2020) described a new reaction in which all components were incubated in a single system without separate preamplification steps. This assay can detect SARS-CoV-2 and HIV-1 simultaneously at 37°C for 40 min. As an innovative attempt, it is able to detect as low as 4.6 copies RNA targets and 1.2 copies DNA targets (Ding et al., 2020). Some studies have shown that HIV infection can alter T cell functions and increase the chance of severe disease in patients with COVID-19 (Riou et al., 2021). So, this technique has potential value for screening critical patients.

In summary, RPA-based assay has the shortest reaction time of all current nucleic acid amplification technology (Amer et al., 2013). Its reaction temperature is around 37°C and can even react at room temperature under certain conditions (Loo et al., 2013). In addition, RPA allows for co-detection of multiple targets, which brings it a wider range of clinical application settings (Ding et al., 2020; El Wahed et al., 2021).

### 2.3 Rolling circle amplification

Rolling circle amplification (RCA) is another rapid and effective isothermal nucleic acid amplification method. Circular DNA template, DNA or RNA polymerase, short primer and deoxy-nucleotide triphosphates (dNTP) or nucleotide triphosphates (NTP) are the main component of the RCA reaction system (Kang et al., 2022). For RNA sequence amplification, *Escherichia Coli* RNA polymerase is essential for the successful running of the reaction (Lizardi et al., 1998). RCA also has the ability to integrate with other emerging technologies like nanobiotechnology and CRISPR (Kang et al., 2022), which allows it to monitor the background level and reduce the false-positive rate (Tian et al., 2020). As for SARS-CoV-2 detection, RCA-based techniques also contribute to the control of the pandemic. Liu et al. (2021) presented a completely new demonstration of the RCA-CRISPR system, which consists of a padlock probe-based RCA step and a subsequent CRISPR-Cas12a-based signal amplification step. The whole reaction takes 3 h at 30°C, with a LOD of 30.3 fM and a good specificity. Detection results of clinical samples (*n* = 48) by this method showed 100% concordance with RT-PCR (Liu et al., 2021). In summary, RCA is an easy and efficient isothermal enzymatic method using unique DNA and RNA polymerases with high specificity.

### 2.4 Nicking enzyme amplification reaction

The reaction system of Nicking Enzyme Amplification Reaction (NEAR) includes three enzymes: reverse transcriptase, isothermal amplification enzyme (Bst), and nicking enzyme. The upstream and downstream primers perform strand displacement amplification on the target sequence under the action of isothermal amplification enzyme, and the nickase could recognize the specific sequence and cut the 8–16 base single strand to form a gap. The gap allows for the continued synthesis of short sequences using isothermally amplified DNA polymerases. The synthesized short-sequence are then combined with fluorescent primers for quantitative analysis. The ID NOW COVID-19 assay is a rapid and easy SARS-COV-2 method via NEAR. The ID NOW COVID-19 assay yielded a sensitivity, specificity, positive prediction value (PPV) and negative prediction value (NPV) of 98.0%, 97.5%, 96.2%, and 98.7% (NguyenVan et al., 2021).

### 2.5 Non-enzymatic isothermal strand displacement and amplification

Since most of the existing nucleic acid amplification techniques rely on biological enzymes, Mohammadniaei et al. (2021) developed a non-enzymatic whole genome detection method named non-enzymatic isothermal strand displacement and amplification (NISDA). This technique adds the displacement and amplification step on the basis of toehold-mediated strand displacement (TMSD) (Yurke et al., 2000; Lee et al., 2020b; Do et al., 2021). One DNA duplex and two DNA molecular beacon structures are the main components of the reaction system. The LoD of this method reaches 10 copies μL^−1^ under the condition of 42°C for 30 min (Mohammadniaei et al., 2021). As for clinical verification, NISDA assay represents 100% specificity and 96.77% sensitivity. This efficient method has generous storage conditions so it is thought to be a very fine complement to RT-PCR (Mohammadniaei et al., 2021).

The isothermal amplification methods could greatly reduce the dependence of detection on equipment. However, this method still has the problems such as the contamination of amplification products and high cost of multiple enzymes. We summarize some isothermal amplification methods in the Table 2; Figure 1.

**TABLE 2:** Isothermal amplification methods for SARS-CoV-2.

	Year	Output method	Isothermal type	Detect time	Detection limit	Advantages and disadvantage
1	2020 (Zhu et al., 2020)	Lateral Flow	RT-LAMP&NP	<1 h	0.48 copies/ul	+ Rapid, sensitive and visible result
						**-** Risk of cross contamination; cost of LAMP&NP
2	2020 (Lu et al., 2020)	Fluorescence	RT-LAMP	<1 h	4.74 copies/ul	+ Simple RT-LAMP assay for the fast and accurate detection of SARS-CoV-2
						- Low sensitive
3	2020 (Qian et al., 2020)	Lateral Flow	RT-RPA&RnaseH	45 min	3–10 copies/test	+ Rapid, sensitive and visible result, unextracted sample
						- Risk of cross contamination; Commercial availability of RT-RPA reagents
4	2021 (El Wahed et al., 2021)	Fluorescence	RT-RPA	15 min	2–15 copies/test	+ The RPA assays were run in a mobile suitcase laboratory to facilitate the deployment at point of need
						- Commercial availability of RT-RPA reagents
5	2020 (Ding et al., 2020)	Fluorescence	RT-RPA&Cas12	40 min	4.6 copies/test	Rapid, sensitive, one-pot reaction and visible result
						- Commercial availability of RT-RPA reagents, high cost of enzyme
6	2021 (Liu et al., 2021)	Glucose	RCA&Cas12	171 min	47p.m.	+ user-friendly, portability
						- Low sensitive, time consuming, cost of RPA & CRISPR reagents
7	2021 (Mohammadniaei et al., 2021)	Fluorescence	NISDA	30 min	10 copies/ul	+ No reverse-transcription step and rapid, affordable, highly robust at room temperature (>1 month), isothermal (42 °C) and user-friendly
						- Low sensitive
8	2021 (Do et al., 2021)	TMB	CHA&Elisa	2 h	1 nM	+ Not require expensive equipment, complex protocols, or long time periods to amplify target DNA
						- Low sensitive, time consuming

## 3 Electrochemistry

Electrochemical biosensors have attracted much attention since they were developed and have been widely used in many fields, mainly focusing on the detection of pollutants in water and pathogenic microorganisms. Based on the specificity of DNA sequences and the principle of complementary base pairing of Watson-Crick, electrochemical nucleic acid biosensors have become an important branch of sensors and play an important role in biomedicine. Such devices work by converting DNA base pair recognition events into useful electrical signals (Wang, 2002). The materials of the sensor components are various according to different experimental designs, but the core is the design of the capture sequence and complementary sequence and the conversion of the signal.

**FIGURE 2:**
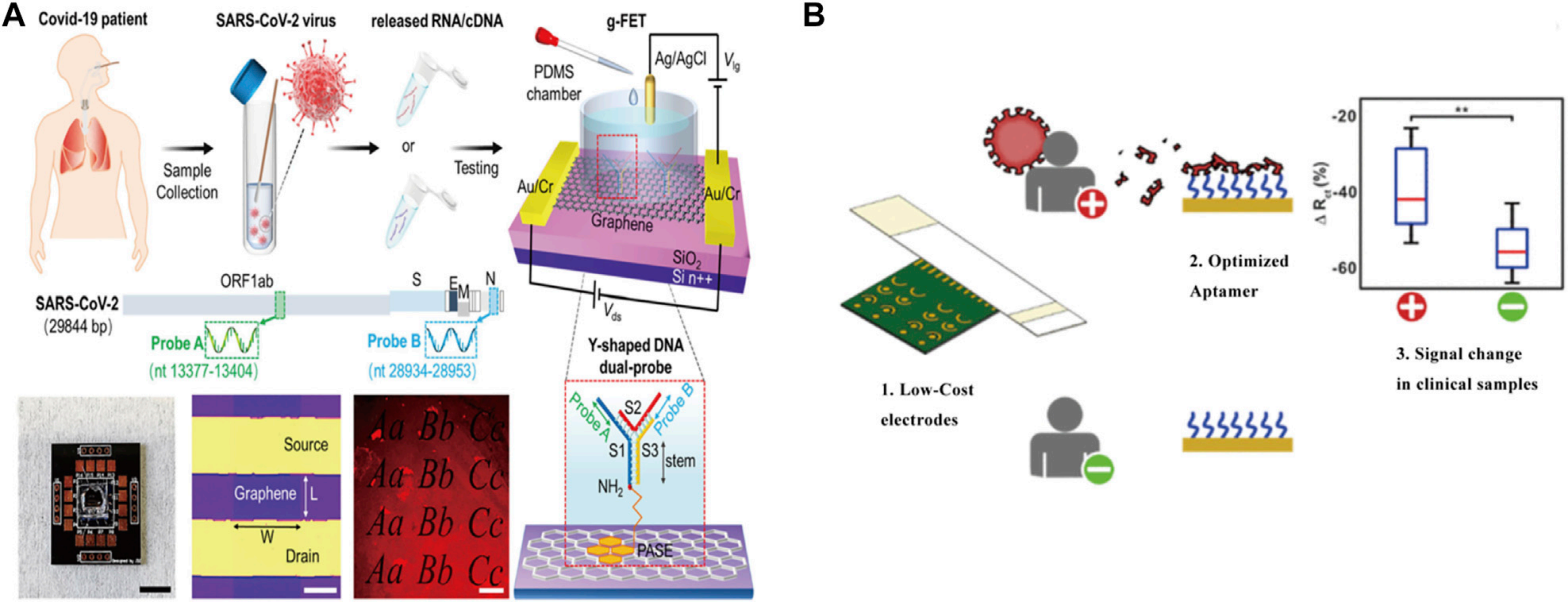
Schematic of Electrochemical detect for SARS-COV-2. **(A)** Direct accounting assay is realized by the design of Y-shaped DNA double probe (Y-dual probe) which is modified on a graphene field effect transistor (gFET) to simultaneously detect ORF1ab and N gene of SARS-CoV-2 nucleic acid. Reprint from (Kong et al., 2021), copyright (2020), with permission from Springer Nature. **(B)** Low-cost and real-time detection of 2019-nCoV by immobilizing an aptamer against the SARS-CoV-2 spike protein on a gold-coated polyester substrate. Reprint from (Lasserre et al., 2022), copyright (2022), with permission from ACS Publications.

Electrochemical sensor is very promising, which brings us a rapid and sensitive POCT detection for SARS-COV-2 detection solution. However, the guaranteed profit in electrochemical sensors in commercial applications depends on the selection of suitable nanomaterials as useful biosensors. In addition, the risk of errors such as false positives in virus detection should be considered when using Reusable and portability nanomaterials. We summarize some electrochemistry methods in the Table 3 and Figure 2.

**TABLE 3:** Electrochemical nucleic acid methods for SARS-CoV-2.

	Year	Pre-amplification	Detect time	Detection limit	Advantages and disadvantage
1	2021 (Zhao et al., 2021a)	no	3 h	200 copies/ml	+1. High sensitivity and sensitivity, low cost, user-friendliness, and robustness; 2. No nucleic acid amplification and reverse transcription
					- Time consuming, device required
2	2021 (Chaibun et al., 2021)	yes	<2 h	1 copy/μL	+1. RCA can be performed under isothermal conditions with minimal reagents and avoids the generation of false-positive results and is less complicated; 2. Combine the high amplification capability of RCA with the sensitivity of the electrochemical detection method
					- Pre-amplification, risk of cross contamination, device required
3	2021 (Kumar et al., 2021)	yes	--	10 pg/μL	+ Inexpensive and reusable
					- Pre-amplification, low sensitivity, risk of cross contamination, device required
4	2020 (Alafeef et al., 2020)	no	<5min	6.9 copies/μL	+1.Rapid, low-cost, easy to implement, and quantitative; 2.An improved limit of detection, no need for additional redox medium for electron exchange, faster response to achieve stable data, excellent shelf life, and its plausible economic production; 3.Overcomes the limitations of widely used antibody-based serological tests, as the developed test can detect the early stage of infection
					- Low sensitivity, device required
5	2022 (Crevillen et al., 2022)	no	<7min	15 × 10^−9^ M	+1.Easy-to-use, disposable and low-cost; 2.Fast detection with minimal sample and reagent consumption
					- Low sensitivity, device required
6	2022 (Deng et al., 2022)	no	--	45 fM	+1.excellent selectivity, good reproducibility, and high resistance in complex environments 2.Low-cost and user-friendly
					- Low sensitivity, device required
7	2022 (Kashefi-Kheyrabadi et al., 2022)	no	<1 h	6.8 ag/μL	+1.Multiplexed detection that avoids the generation of false negative results; 2.High specificity and ability to differentiate between closely related RNA target sequences down to single nucleotide substitution; 3.A single step procedure and short assay period; 4.Low LOD that satisfies sensitivity requirement and could potentially be used to detect SARS-CoV-2 RNA targets in the early stages of the disease while the viral genes load is low
					- Device required
8	2021 (Farzin et al., 2021)	no	--	0.3 p.m.	+ A wide linear range of detection, low LOD, and good selectivity
					- Low sensitivity, device required
9	2021 (Peng et al., 2021)	no	--	26 fM	+1. The strong anti-interference ability and accuracy; 2. Detect the SARS-CoV-2 RNA in different samples with excellent stability; 3. Simple, low-cost and easy-to-operate
					- Low sensitivity, device required
10	2021 (Zhao et al., 2021b)	no	<20 min	7 copies/μL	+1. Streamlines the assay workflow and improves the system robustness.; 2. Automate the assay workflow and have high sensitivity
					- Device required

## 4 CRISPR system

**FIGURE 3:**
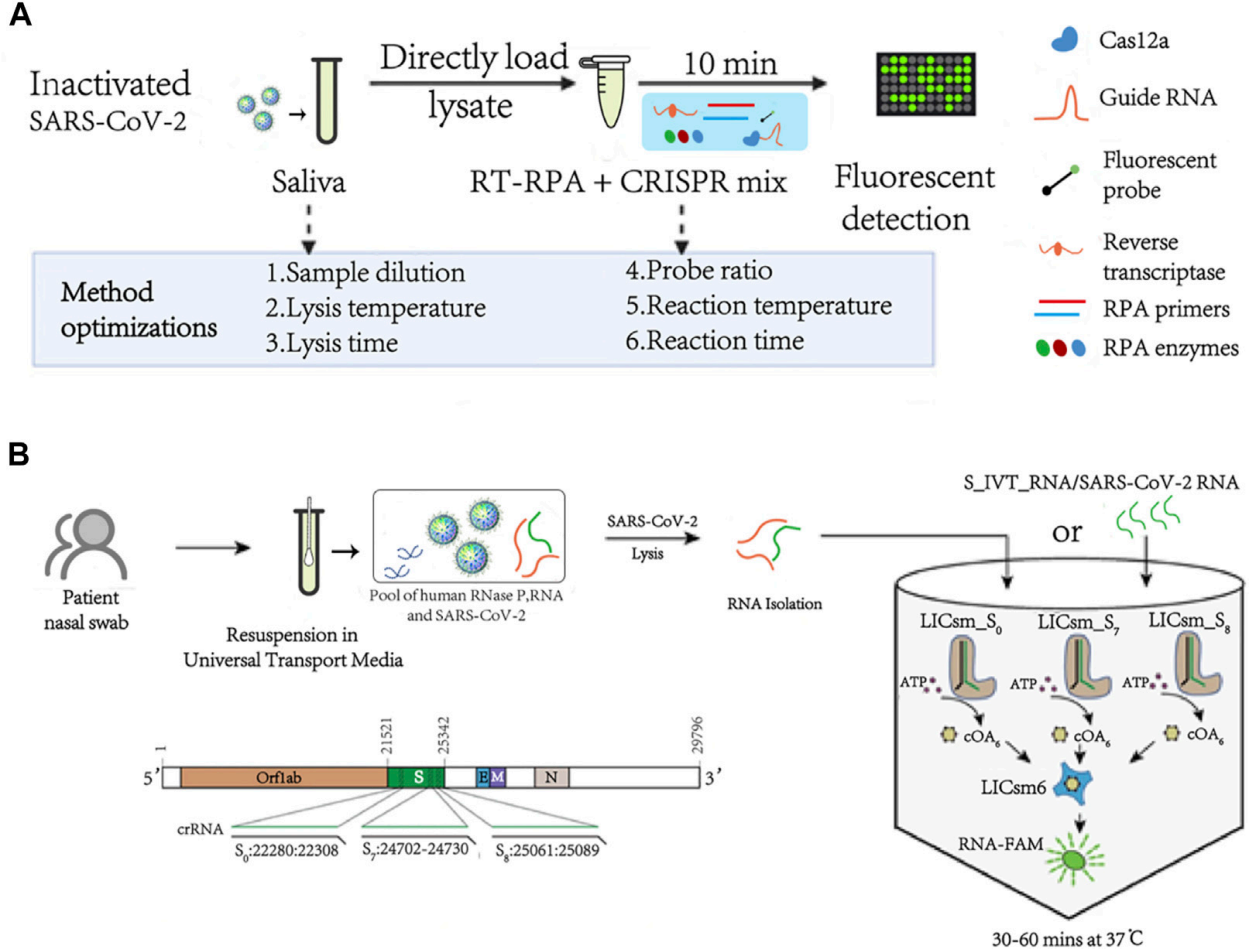
Schematic of CRISPR-Cas methods for SARS-COV-2. **(A)** Optimization workflow for RNA recovery and CRISPR-FDS reaction efficiency. Reprint from (Ma et al., 2022), copyright (2021), with permission from AAAS. **(B)** Workflow of MORIARTY detection of SARS-CoV-2 through a multiplex one-pot reaction strategy and schematic SARS-CoV-2 genome harboring Orf1ab, S, E, M, and N genes. Reprint from (Sridhara et al., 2021), copyright (2021), with permission from Springer Nature.

CRISPR (the clustered regularly interspaced short palindromic repeats) and CRISPR–Cas (clustered regularly interspaced short palindromic repeats–CRISPR-associated proteins) are adaptive immune systems in archaea and bacteria, bringing great changes to the development of gene editing technology (Barrangou et al., 2007; Feng et al., 2021). CRISPR–Cas systems use Cas protein as endonuclease to recognize and cleave specific nucleic acid sequences under the guidance of single-guide RNA (Abudayyeh et al., 2016). Based on the high specificity and high sensitivity of the CRISPR-Cas system, the detection technology of CRISPR-Cas has been rapidly developed and applied to the next-generation nucleic acid detection technology. CRISPR-Cas-based diagnostic technologies mainly use Cas12 and Cas13 enzymes, which can target DNA or RNA and possess collateral DNase or RNase activities (Freije and Sabeti, 2021). Besides, this system also has great potentials in biosensing devices that can be used for SARS-CoV-2 detection (Morales-Narvaez and Dincer, 2020). Long Ma et al. reported a CRISPR-Cas12a powered visual biosensor for ultrasensitive detection of SARS-CoV-2 (Ma et al., 2022). Fozouni et al. reported the development of an amplification-free CRISPR/Cas13a assay for detecting SARS-CoV-2 directly from nasal swab RNA that can be read with a mobile phone microscope (Fozouni et al., 2021). Kai Zhang et al. constructed an exonuclease III cleavage reaction-based isothermal amplification of nucleic acids with CRISPR/Cas12a-mediated pH-induced regenerative Electrochemiluminescence (ECL) biosensor for detection of SARS-CoV-2 nucleic acids (Zhang et al., 2022). In general, the CRISPR-Cas system has good specificity and sensitivity, and has good application prospects in the detection of viruses such as SARS-COV-2. However, the pretreatment of some CRISPR detection systems still relies on amplification, which may lead to problems such as product contamination and high cost of detection. Most of these methods are visualized, fast and sensitive, as listed in Table 4; Figure 3.

**TABLE 4:** CRISPR detection system for SARS-CoV-2.

	Year	Cas type	Detect time	Detection limit	Advantages and disadvantage
1	2022 (Lu et al., 2022)	Cas13a	1 h	1 copies/ml	+ High sensitivity, specificity, and repeatability, without complicated operation and expensive equipment
					- Device required, Pre-amplification
2	2021 (Fozouni et al., 2021)	Cas13a	30 min	100 copies/ml	+ Rapid, low-cost, point-of-care screening, quantitative, accurate, simplicity and portability
					- Device required
3	2022 (Zhang et al., 2022)	Cas12a	2–3 h	43.70a.m.	+ Stability, reproducibility and high sensitivity
					- Device required, time consuming
4	2022 (Wang et al., 2022b)	Cas13a	25 min	3 copies/ul	+ Rapid, simple, low-cost, complete the nucleic acid detection without opening the lid, no need for any specialized equipment
					- Pre-amplification, cost of RPA & Cas reagents
5	2022 (Lu et al., 2022)	Cas12a	<20 min	1 copies/ul	+ One step, fast speed, high sensitivity, high reliability and flexibility, no need for RNA extraction, as sensitive、reliable and flexible as RT–qPCR, can be visualized with naked eyes
					- Pre-amplification, cost of RPA & Cas reagents
6	2022 (Ma et al., 2022)	Cas12a	<90 min	1 copies/ul	+ Ultrasensitive, specific, simple and visualized, no cross-reactivity, provides a novel and robust technology for ultrasensitive detection
					- Pre-amplification, time consuming
7	2022 (Fozouni et al., 2021)	Cas13a	<30 min	0.6copies/ul (inactive) 1.38 copies/ul	+ Ultrasensitive, amplification-free, adapted to detect a variety of nucleic acid targets for medical diagnostics, environmental monitoring, and food safety
					- Device required
8	2022 (Wang et al., 2022b)	Cas13a	2–3 h	0.216 fM	Quantification, high specificity, discrimination of highly homologous coronaviruses, discriminate single-nucleotide mutation, not involve labeling probes and expensive technical equipment
					- Cost of T4 T7 & Cas reagents, time consuming
9	2021 (Ning et al., 2021)	Cas12a	15 min	0.38 copies/ul	+ Portable, ultrasensitive, not require RNA isolation or laboratory equipment
					- Cost of RPA & Cas reagents
10	2021 (Sridhara et al., 2021)	Cas Type III-A	30 min	2000 copies/ul	+ Rapid reporting, high sensitivity, flexible reaction conditions, and the small molecular-driven amplification
					- Low sensitivity

## 5 Nucleic acid enzymes

**FIGURE 4:**
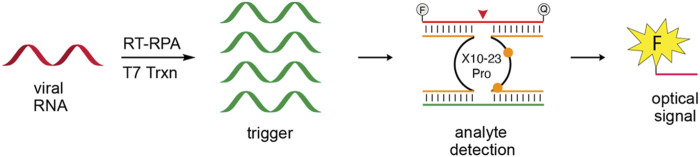
Schematic of XNAzyme methods for SARS-COV-2. Reprint from (Yang and Chaput, 2021) copyright (2021), with permission from ACS.

The development of *in vitro* selection methods, such as Systematic Evolution of Ligands by Exponential Enrichment (SELEX), to screen DNA or RNA from random sequence libraries has led to the development of further study of synthetic nucleic acids with special properties (Tuerk and Gold, 1990). These new DNA or RNA sequence offer more stable, and less expensive choice for detection platforms (McConnell et al., 2020). DNAzymes are synthetic ssDNA oligonucleotides with specific catalytic abilities (Silverman, 2010), designed for the detection of SARS-CoV-2 RNA. This design was highly sensitive (10^3^ copies of viral RNA) for the N gene of SARS-CoV-2, which is not present in other viruses (Anantharaj et al., 2020). Yang reported a novel platform a detection limit of ≤20 a.m. for SARS-COV-2 in 1 h with a XNAzyme 10–23 (Figure 4), (Yang and Chaput, 2021). Compared with CRISPR system, nucleic acid enzyme has the advantages of no PAM motif, no protein expression and low cost.

## 6 Summary and perspective

The ongoing epidemic of SARS-COV-2 has severely impacted the global economy, travel, work and living habits. Recovery from the social and economic impact of SARS-CoV-2 has been prolonged due to differences in virus control measures in different countries (Kevadiya et al., 2021). Therefore, there is still a continuing demand for the diagnosis of SARS-CoV-2. The development of various new technologies brings us potential methods for better and faster viral nucleic acid detection (Li et al., 2021; Song et al., 2022). Most importantly, PCR-free virus detection techniques facilitate the development of sensitive, simple, scalable, rapid, and cost-effective detection methods for SARS-CoV-2, which could be translated to the development of rapid detection systems for the early response to various other infectious diseases, including newly found viruses, waste water-based epidemiology *etc*. However, few studies take full advantage of PCR-free methods in clinical translation. Many new methods always have some problems, such as instrument-dependent, expensive, and poor product reproducibility. Only assays that are sensitive, simple, rapid, reproducible, and cost-effective could be used in clinical translation.

In this review, we summarized methods of isothermal amplification, electrochemistry, CRISPR, and SERS methods developed for SARS-CoV-2 detection including the underlying scientific principles. We hope this review article would help the readers have a basic understanding of the current state of non-PCR methods for SARS-CoV-2 detection.
